# Staggered Design of UV–Curable Polymer Microneedle Arrays with Increased Vertical Action Space

**DOI:** 10.3390/polym17010104

**Published:** 2025-01-02

**Authors:** Baoling Jia, Tiandong Xia, Yangtao Xu, Bei Li

**Affiliations:** 1 School of Materials Science and Engineering, Lanzhou University of Technology, Lanzhou 730050, China; 2 State Key Laboratory of Advanced Processing and Recycling of Non-Ferrous Metal Under the Province and the Ministry of Education, Lanzhou University of Technology, Lanzhou 730050, China

**Keywords:** microneedle, various heights, microneedle penetration, 3D printing

## Abstract

Recent studies have identified microneedle (MN) arrays as promising alternatives for transdermal drug delivery. This study investigated the properties of novel staggered MN arrays design featuring two distinct heights of MNs. The staggered MN arrays were precisely fabricated via PμSL light-cured 3D printing technology. The arrays were systematically evaluated for their morphology, fracture force, skin penetration ability, penetration mechanism, and drug delivery capability. The results demonstrated that the staggered MN arrays punctured the skin incrementally, leveraging the benefits of skin deformation during the puncture process. This approach effectively reduced the puncture force needed, achieving a maximum reduction of approximately 80.27% due to variations in the staggered height. Additionally, the staggered design facilitated skin penetration, as confirmed by the results of the rat skin hematoxylin-eosin (H&E) staining experiments. Compared with 3D-printed planar structures and highly uniform MN arrays, the staggered design exhibited enhanced hydrophilicity, as evidenced by a reduction in the contact angle from approximately 93° to 70°. Simulated drug release images of both coated and hollow staggered MNs illustrated the release and delivery capabilities of these structures across various skin layers, and the staggered design expanded the effective area of the MN arrays within the vertical dimension of the skin layers. This study offers both experimental and theoretical foundations for developing MN arrays with three–dimensional structural distributions, thereby facilitating advancements in MN array technology.

## 1. Introduction

MN technology has emerged as a method capable of penetrating the stratum corneum and reaching the epidermis without affecting the nerve endings in the dermis. This technology offers a painless, convenient, and highly effective alternative for applications such as cosmetic enhancement, transdermal drug delivery, and monitoring. During the global COVID-19 pandemic, MN technology had advantages such as the absence of expensive equipment or extensive training. It can facilitate testing and treatment in underserved areas, potentially expediting health restoration. Consequently, MNs technology has generated considerable anticipation and has been recognized as one of the top 10 emerging technologies of 2020 [1].

MNs are made from various raw materials, utilize multiple preparation methods, exhibit unique morphological designs, and have diverse applications. Many studies have shown an intrinsic relationship between the functionalization applicability of MNs and their morphological design [2,3]. For example, to improve the drug-carrying capacity of MNs, researchers can design MNs with greater heights or larger tip sizes. However, increasing the height may result in increased pain, and enlarging the tip size can lead to increased difficulty in MN penetration of the skin. Consequently, establishing a balanced trade-off between the drug-carrying capacity and the morphological design of the MNs is crucial. Four key factors are generally considered when designing the morphology of MNs: (1) their ability to penetrate the skin; (2) the mechanical strength needed to withstand bending, breaking, and flexing; (3) the feasibility of machining and manufacturing processes; and (4) the potential to improve the efficiency of functionalized applications. To comprehensively meet these requirements, the morphological design of MN arrays should prioritize not only the shape and size of individual MNs but also the arrangement of all MNs within the whole array patch.

The morphology of individual MNs has attracted considerable interest from researchers. Advanced processing technology and innovative thinking provide opportunities to diversify the shapes of MNs. Most MNs are conical or pyramidal in geometric shape. The primary advantage of these shapes is their ease of preparation, and they can be fabricated using common micro–molding methods, 3D printing, or plate stretching. Nevertheless, certain specialized geometric shapes can enhance the functional performance of MNs. Arrow–shaped MNs can accommodate the elasticity of the skin, improving the penetration depth [4]. Turret-shaped MNs have a pointed tip and a broad base, which enhance mechanical strength and facilitate skin penetration [5]. Faceted MNs have a larger surface area than smooths of equivalent size, resulting in increased drug-loading capacity in the drug model layer [6].

Researchers have highlighted the importance of MN dimensions, including height, base diameter, tip size, and sharpness. The painlessness of using MNs is primarily attributed to their limited height, which typically ranges from 600 to 1000 μm. This dimensional range guarantees a pain-free and bleed-free application process, facilitating the penetration of the epidermal layer for drug delivery and subcutaneous monitoring. If the MN is excessively short, it cannot puncture the skin; conversely, if it is excessively tall, it may cause pain and reduce mechanical strength. The increase in the diameter of the MN base reduces its displacement within the skin, minimizes the risk of fracture, and increases the drug-loading capacity. The size of the MN tip is crucial for effective skin penetration. The penetration force is linearly correlated with the interfacial area of the MN tip. A tip that is too short or blunt may cause the skin to fold, impeding insertion during epidermal penetration. Research has indicated that relatively blunt MN tips with diameters ranging from 60 to 160 µm necessitate relatively high penetration forces (0.08–3.04 N) [7].

A single MN has limited drug capacity and efficacy; thus, multiple MNs are organized into a square or a circular array. The spacing between the tips, the MN density, and the backing layer design are crucial factors affecting MN arrays. Owing to the viscoelastic properties of the skin, contact with the MNs causes skin deformation and contraction. High-density MNs require greater force to achieve significant penetration, potentially causing increased soreness for patients or necessitating additional equipment for assistance. While smaller spacing between MN tips or a higher density increases the drug load and contact area with interstitial fluid, neighboring MNs can interact, creating a “nail bed effect” that prevents MNs from penetrating the skin [8]. Increasing the spacing between MN tips decreases the penetration force and enhances the MN penetration ratio, facilitating improved drug transmission [9]. However, excessively large tip spacing requires a larger backing layer and insertion aid, thus increasing the overall dimensions of the MN device. The MN density and tip spacing are interrelated and should be maintained within a reasonable range.

Conventional MN arrays typically consist of a uniform shape and size designed to facilitate synchronized and effective skin penetration. However, some studies have found that the MN arrays had the behavior of deep piercing at the edge and shallow piercing at the center during skin piercing [10], and the thickness and flexibility of the backing layer of the MN patch can influence the overall penetration behavior. Previous studies have statistically indicated that the effectiveness of MN arrays in penetrating skin tissue has not yet achieved satisfactory results. Most MNs only penetrate to a depth of 30–60% of their total length. Furthermore, enhancing effective MN penetration remains a primary focus in ongoing developments in MN technology.

Designing MNs with varying densities and heights within the same array is anticipated to reduce the overall puncture force and address the issue of puncture depth. During the puncture process, MNs of varying heights sequentially penetrate the stratum corneum. This sequential penetration leads to a reduction in both the puncture force of the array and the associated tingling sensation. Compared with the periphery, the MN array features a lower density of MNs in the central region, which facilitates the stable and low insertion of the MNs into the skin while increasing the total number of MNs in the array [10]. Johnson et al. [5] utilized continuous liquid interface production, enabling the rapid preparation of tiered-structured MNs. Patent [11] outlined a method for producing high-density, asymmetric crystalline MN arrays. This invention permits the creation of MNs with varying heights through adjustments to the mask shape and size. Another patent also details a technique that combines wet etching with mechanical processing to develop stepped MN arrays [12]. This suggests that incorporating MNs of differing heights into a single array is feasible from a manufacturing perspective. Furthermore, MNs of varying heights can deliver drugs to specific locations within different layers of the skin. For example, in chronic wound applications, shorter MNs target the epidermis, whereas longer MNs penetrate the dermis to release their payload into deeper layers [13]. Likewise, varying height designs also facilitate access to physiological signals at various vertical and horizontal locations during MN percutaneous monitoring [14].

The variable height structure introduces an innovative design strategy intended to enhance the performance of MN arrays. However, not all MN arrays that utilize this design efficiently penetrate skin tissue. Hu [15] designed MNs in two configurations: a single row and multiple rows of unequal height MNs with a height difference of 0.34 mm. The single row of MNs effectively addresses the issue of asynchronous puncturing, whereas the multiple-row MN arrays do not achieve this outcome.

This study successfully fabricated staggered structured UV-curable polymer MN arrays with various morphological designs via PμSL light-cured 3D printing technology. We investigated the mechanical properties of these MN arrays, which included fracture force, puncture force on porcine skin, and in vitro rat skin puncture depth, along with the puncture process. Finally, we evaluated the hydrophilicity and in vitro drug release properties of the staggered MN arrays, offering valuable insights for their future applications.

## 2. Materials and Methods

### 2.1. Materials and Animals

Biocompatible photosensitive resin (BMF Precision Technology Co., Ltd., Shenzhen, China), chloral hydrate (C8383, Sigma, Darmstadt, Germany), general-purpose tissue fixative (B0038, Wuhan Powerful, Wuhan, China), LPS (L8880, Solarbio, Beijing, China), PBS (P1020, Solarbio), acetone (10000418, Sinopharm Group, Beijing, China), erythrocyte lysate (SR775701, Sinopharm Group), lymphocyte isolation (P8620, Solarbio), anhydrous ethanol (100092683, Sinopharm Group), xylene (10023418, Sinopharm Group), HE staining kit (BH0001, Wuhan Powerful), hematoxylin differentiation solution (B0020, Wuhan Powerful), hematoxylin back to blue solution (B0021, Wuhan Powerful), porcine skins (local markets), rhodamine B (C15205925, Macklin, Shanghai, China), agarose powder (C16154274, Macklin), Nafion (5%, Sigma), pigment, and deionized water.

Research involving animal procedures was performed according to the Guide for the Care and Use of Laboratory Animals, relevant policies in China, and the Guidelines of the Biomedical Ethics Committee of the School of Life Sciences and Engineering at Lanzhou University of Technology. The rats were obtained from the Hubei Experimental Animal Research Center, and animal experiments were conducted at Kangtai Medical Laboratory Service Hebei Co., Langfang, China.

### 2.2. Instruments and Equipment

An additive manufacturing system (PμSL light-cured, microArch S240, BMF Precision Technology Ltd., Shenzhen, China), a digital microscope (VMS700 Pro, Shenzhen, China), China), a thermal field emission scanning electron microscope (Quanta FEG 450, FEI Company, Hillsboro, OR, USA), a texture analyzer (TA. XTC-18, Shanghai Prosun Industrial Development Co., Ltd., Shanghai, China), a dehydrator (JT-12J, Wuhan Junjie Electronics Co., Ltd., Wuhan, China), an embedding machine and freezing table (JB-L5, Wuhan Junjie Electronics Co., Ltd., China), a tissue spreader machine (JK-6, Wuhan Junjie Electronics Co., Ltd., China), an oven (DGX–9003B, Shanghai Formosa Experimental Instrument Co., Ltd., Shanghai, China), an orthostatic optical microscope (Nikon Eclipse CI, Tokyo, Japan), an imaging system (FI3, Nikon, Tokyo, Japan), a contact angle–measuring instrument (SL250, KINO Scientific Instrument. Inc., Boston, MA, USA), and a vacuum plasma processor (NE-PE02, NAEN Technology, Shenzhen, China) were used.

### 2.3. Design and Additive Manufacturing of MN Arrays

**Figure 1:**
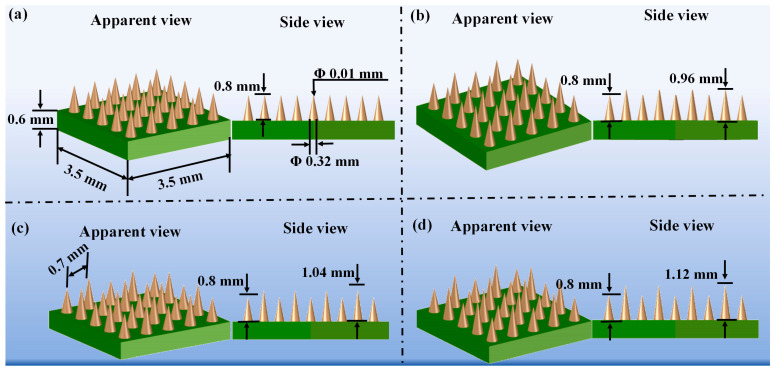
Arrangement of the MNs in array patches: (**a**) #1 MN arrays with a height of 0.8 mm; (**b**) #2 MN arrays with heights of 0.8 mm and 0.96 mm; (**c**) #3 MN arrays with heights of 0.8 mm and 1.04 mm; and (**d**) #4 MN arrays with heights of 0.8 mm and 1.12 mm.

Four MN arrays were designed via AutoCAD 2023. Each array contained 25 MNs arranged in a 5 × 5 rectangular array, with a patch size of 3.5 mm × 3.5 mm × 0.6 mm. The standard MN arrays (Figure 1a) consisted of 5 × 5 conical MNs, each measuring 0.8 mm in height, with a base diameter of 0.32 mm and a tip diameter of 0.01 mm. The interspacing between the MNs was 0.7 mm, and the backing layer had a thickness of 0.6 mm. The staggered MN arrays (Figure 1b–d) featured two different heights. The shorter MNs maintained the same dimensions as the standard array, whereas the longer MNs were designed with heights of 0.96 mm, 1.04 mm, and 1.20 mm, a base diameter of 0.32 mm, and a tip diameter of 0.01 mm. The two types of MNs were arranged with a tip interspacing of 0.7 mm. Figure 1 illustrates the arrangement of the MNs in the staggered structure array patches.

MN arrays were fabricated from light–curing resin via the face-projected microscale high–precision light–curing additive manufacturing (PμSL) technique. The CAD-designed 3D models were exported in the STL format, and the print patterns were generated by slicing software (Magics, Customized version). The biocompatible photopolymer resin was cured and shaped by an additive manufacturing system under 405 nm ultraviolet light, achieving a printing accuracy of 10 μm. The models were rinsed with ethanol and distilled water, followed by drying.

### 2.4. Characterization of Staggered MN Arrays

#### 2.4.1. Morphology

Digital microscopy was utilized to examine the macroscopic morphology of the MNs. SEM at a voltage of 10 kV was used to observe the microscopic morphology of the MNs.

#### 2.4.2. Fracture Force

**Figure 2:**
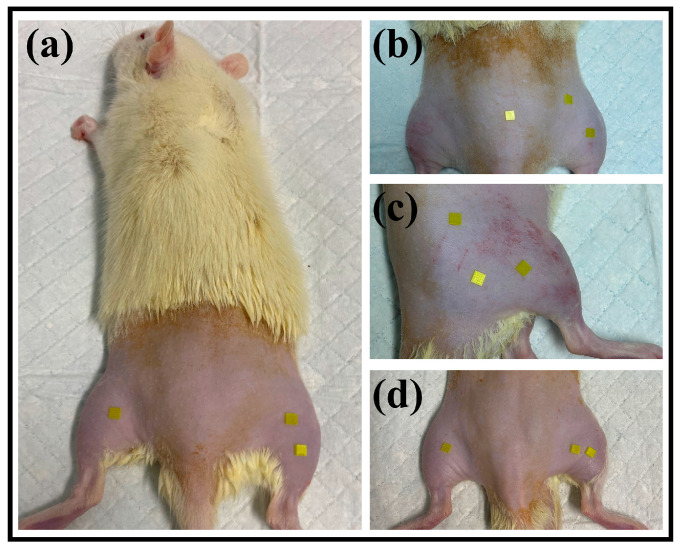
Photographs of MN patches puncture rats: (**a**) #1 MN arrays; (**b**) #2 MN arrays; (**c**) #3 MN arrays; (**d**) # 4MN arrays.

The fracture force of the MNs was assessed via a texture analyzer. The testing methodology is presented in Figure 2a. The MN arrays were positioned on the lower workbench, and the upper working probe (stainless steel probe, 5 mm diameter) was moved toward the MNs at a speed of 0.02 mm/s. The trigger force was set to 0.05 N, and the downward displacement was 0.4 mm. Load and displacement measurements were taken every 0.01 s to create load–displacement curves.

#### 2.4.3. Penetration Force Testing on Porcine Skin

Porcine skin samples were acquired from local markets. The testing schematic is shown in Figure 2b. Following the removal of connective tissue using a scalpel, the skin was placed flat on the test bench surface. The penetration force of the MNs was evaluated using a texture analyzer. The MN arrays were positioned on the porcine skin, and the upper working probe was moved toward the MNs at a speed of 0.01 mm/s, resulting in the generation of a load–displacement curve.

#### 2.4.4. Penetration Depth Test in Rat Skin

**Figure 3:**
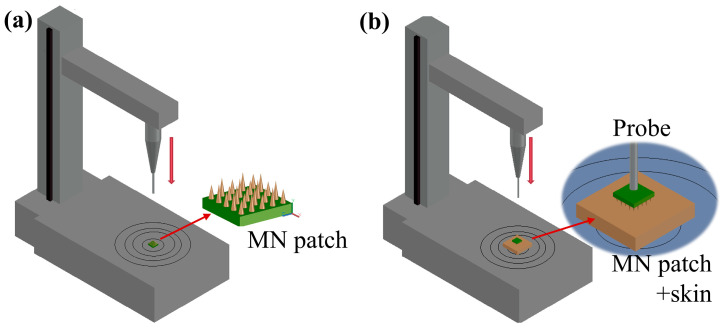
Schematic of the mechanical properties of the MN patches: (**a**) the fracture force, and (**b**) penetration force test on porcine skin.

MN arrays of varying heights were inserted into the dorsal skin of each rat to assess the puncture depth. Photographs of the puncture site are displayed in Figure 3. Eight-week-old male SPF-grade SD rats in good health were utilized. The rats were acclimatized for seven days and monitored for their overall health. The dorsal fur of each rat was shaved to facilitate treatment. MNs specific to each experimental group were applied to the shaved area on the rat backs of each rat and pressed for two minutes before removal. The rats were anesthetized and secured to a dissection table. The target skin samples were carefully excised to ensure that the skin was intact. The histological analysis involved processing the skin samples for paraffin embedding, drying, sectioning, dewaxing, and H&E. Imaging was performed via a Nikon orthogonal light microscope.

#### 2.4.5. Skin Penetration Process

The color–coated MNs were pressed on the surface of the porcine skin. The pictures of the skin were assessed before and after MN insertion via a digital microscope.

#### 2.4.6. Hydrophilicity

The initial static contact angles of deionized water on the MN array surface were measured using a contact angle-measuring instrument. The stop-drop method with a droplet volume of approximately 5 μL was utilized. Data processing was performed via the CAST 3.0 system, and fitting was carried out via the Young–Laplace equation.

#### 2.4.7. Drug Release to Various Skin Layers

Preparation of coated MNs: Rhodamine B served as the model drug, and a suspension was created by dispersing it in a 5% Nafion solution. The MNs were subjected to hydrophilic treatment in a vacuum plasma processor for 15 s. A suspension was applied to the surface of the MNs by dipping, and the coated MNs were allowed to dry in air.

Preparation of hollow MNs: Using the MN array designed in Section 2.3 as a prototype, micro holes with a diameter of 0.08 mm and a dislocation distribution were created on the MN. Additionally, grooves measuring 3 × 3 × 0.3 mm were designed on the backing layer. The entire setup was fabricated via a 3D printer.

Agarose gel preparation: Agarose powder was mixed with TAE buffer, heated in a microwave oven, stirred until fully dissolved, and subsequently poured into a Petri dish for solidification. Agarose gel was used to simulate skin.

Drug release to various skin layers: Absorbent papers, which were impregnated with pigment, were placed into a groove within the backing layer of the hollow MN arrays. The hollow MNs were inserted into the agarose gel, and the agarose gel slices were photographed at different times, respectively, to observe the layered release of the drug in the simulated skin.

### 2.5. Statistical Analysis

All the data are presented as the mean ± standard deviation (SD) of more than three experiments. All the statistical results presented were analyzed via one-way analysis of variance using Origin 2021 software.

## 3. Results and Discussion

### 3.1. Morphology of Staggered MN Arrays

Common MN arrays typically consist of multiple MNs that have the same identical shapes and dimensions, as exemplified by the #1 MN arrays. This study presents a novel array configuration that features two types of staggered MNs with varying heights. The height difference is conceptualized as the puncture displacement of MNs following their complete penetration of the corneal and epidermal layers. Consequently, the #2 MN arrays comprise two sizes of MNs with heights of 0.8 mm and 0.96 mm. The #3 and #4 MN arrays retain the 0.8 mm MNs while introducing additional MNs measuring 1.04 mm and 1.12 mm. We designed the MN patches as a 5 × 5 array, allowing us to emphasize both the advantages and disadvantages of the staggered structural design while simultaneously reducing fabrication costs.

**Figure 4:**
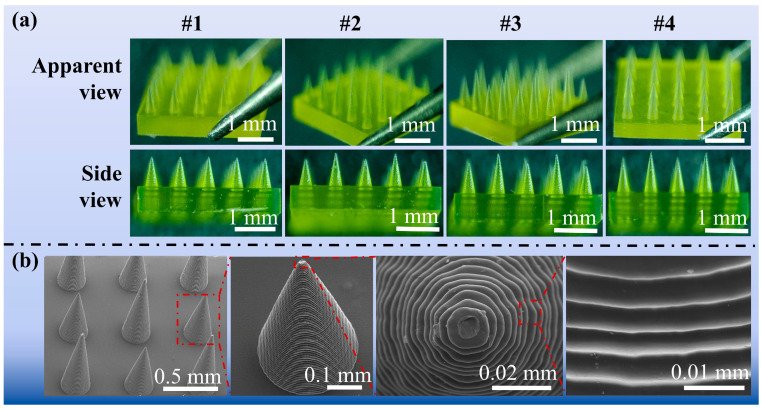
Morphology of the MN arrays: (**a**) digital microscope images and (**b**) SEM micrographs.

The morphological design of the #1 MN arrays serves as a foundational structure in the literature, supported by numerous studies demonstrating its utility. In the design of the staggered structure MN arrays (#2–4), the MNs on the first level are identical to those in the #1 MN arrays. However, the heights of the MNs on the second level vary. Figure 4 shows the morphology of the staggered MN arrays as observed under a digital microscope and scanning electron microscope, highlighting the morphological differences across various viewing angles and magnifications. As depicted in Figure 4a, the prepared MNs exhibit a yellowish color, possess an excellent appearance, demonstrate good size consistency, and display an even distribution. Figure 4b shows the SEM images of the MNs. The results indicate that the resin was cured in layers via an advanced additive manufacturing system, which displayed a ring-mounted pattern under SEM. Additionally, the prepared MN tips were approximately 10 μm in size, with layer heights ranging from 3 to 5 μm.

The design of the MNs, which maintain a uniform diameter at both the top and bottom, indicates that variations in height influence the aspect ratio. SEM observations revealed that the printed MNs were arranged vertically. Furthermore, the layer thickness remains consistent across the different heights and components of the MNs, measuring between 0.8 mm and 1.12 mm. The layer height does not significantly affect the overall height of the MNs. Comparable experimental results have been documented in the literature [16]. The preparation process illustrates the feasibility of producing staggered MN arrays through advanced additive manufacturing techniques.

### 3.2. Mechanical Properties of the Staggered MN Arrays

**Figure 5:**
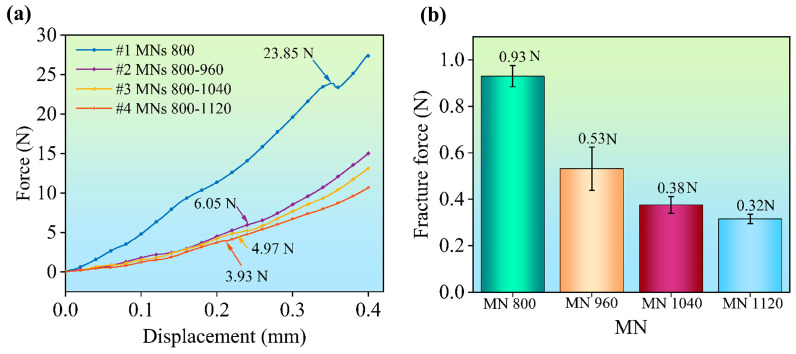
Mechanical compression tests of MN arrays: (**a**) fracture force of MN arrays and (**b**) fracture force of individual MNs.

The mechanical properties of the MN arrays were examined via performing compression tests using a texture analyzer. Figure 5 presents the results of the compression experiments for the four structural designs of the MN arrays. The displacement–force curve identified a flexion point where the force decreased. The 5 mm diameter probe could compress all of the 25 MNs in the #1 MN arrays. The force-displacement curve revealed an inflection point at 0.35 mm, with a total fracture force of 23.85 N and an average individual MN fracture force of 0.93 N. In the #2, #3, and #4 MN arrays, there were 12 high MNs and 13 low MNs, which were arranged in a staggered pattern. Therefore, the critical factor influencing the fracture force in these staggered arrays is whether the high MNs in the second level can withstand the applied response forces. The force–displacement curve for the MN arrays #2–#4 illustrates the fracture resistance of the MNs with greater heights within the array. The total fracture forces for the MNs exhibiting greater heights in the three arrays were 6.05 N, 4.97 N, and 3.93 N, respectively.

Previous studies have reported that cross–linked gelatin methacryloyl MNs can withstand a compressive force of 0.08 N, whereas individual drug/resin MNs withstand a force of approximately 0.26 N per MN [17,18]. The conventional MNs developed in this study demonstrate a single MN breaking force of approximately 0.93 N ± 0.05 N. The staggered MNs demonstrated single MN breaking forces of approximately 0.53 N ± 0.05 N, 0.38 N ± 0.05 N, and 0.32 N ± 0.05 N, indicating sufficient fracture strength. For MNs with the same base width and tip size, those with a height of 1.12 mm are more prone to fracture than those with a height of 0.8 mm, which aligns with the findings reported in the literature [19]. Therefore, pursuing a design for staggered MN arrays with increased height should not come at the expense of basic mechanical properties.

### 3.3. Penetrating Force of the Staggered MN Arrays

**Figure 6:**
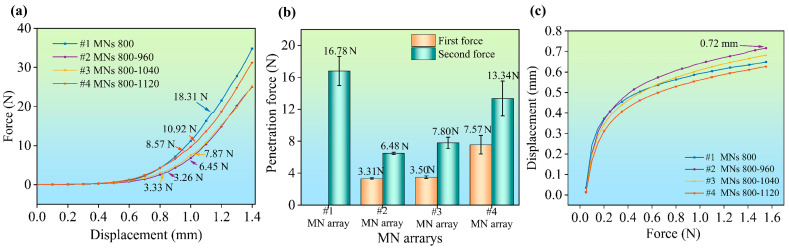
Force diagram of MN arrays puncturing porcine skin: (**a**) penetration force curves of MN arrays; (**b**) penetration force of MN arrays; and (**c**) displacement curves of MN arrays.

A lower penetration force of the MN arrays facilitates insertion into the skin, reduces stress transmission to the subcutaneous nerve tissue, and decreases the likelihood of pain perception. Therefore, assessing the penetration force of staggered MNs for skin puncture is essential. In this study, porcine skin was used as a model for simulating human skin. Figure 6a depicts the displacement force curves observed during the puncture process. The force at the inflection point on the curve indicates the puncture force of the MN arrays, while the corresponding displacement reflects the puncture displacement. The slope of the curve, which represents the relationship between the puncture force and displacement, is defined as the puncture stiffness. The figure shows that the stiffness of the four MN arrays was comparable during the initial puncture stage. However, as the MN arrays penetrated deeper into the porcine skin, the four curves began to diverge, indicating variations in MN stiffness. The total puncture force exerted by the #1 MN arrays remained consistently greater throughout the puncture process, whereas the forces for #2 and #3 were comparable, with the force for #4 falling in between. Notably, a puncture force of 18.31 N was recorded at a displacement of 1.15 mm for the #1 array, which was lower than the total fracture force of 23.85 N. The force-displacement curves of the remaining three staggered MN arrays exhibited two inflection points, indicating that the staggered structural design of the MN arrays facilitated a gradual puncture process in the simulated skin.

The force at the inflection point on the curve represents the puncture force of the MN array, whereas the associated displacement indicates the puncture displacement. The slope that represents the relationship between the puncture force and displacement at each moment is defined as the puncture stiffness. The figure shows that the stiffness of the four MN arrays was similar during the initial puncture stage. However, as the MNs penetrated deeper into the porcine skin, the four curves diverged, revealing differences in MN stiffness. The total puncture force of the #1 MN arrays was consistently greater throughout the puncture process, while the forces for #2 and #3 were comparable, and the force for #4 fell between those of the other arrays. Figure 6b shows the average puncture force of the MN array during successive skin punctures. The total puncture force for the #1 MN arrays is 16.78 N ± 1.8 N. In comparison, the staggered MN array has total puncture forces of 3.31 N ± 0.2 N, 3.5 N ± 0.2 N, and 7.57 N ± 1.1 N. The second stage has puncture forces of 6.48 N ± 0.1 N, 7.8 N ± 0.7 N, and 13.34 N ± 2 N, respectively.

Figure 6c illustrates the variation in displacement resulting from puncturing pig skin under a consistent force. In the initial stress phase, the puncture displacements exhibited by various designed MN arrays are similar. However, as the puncture force increases, the staggered MN arrays significantly increase the degree of puncture displacement. Notably, the #2 MN arrays demonstrate the highest puncture displacement among all the tested arrays. At a force of 1.55 N, the puncture displacement measured approximately 0.72 mm, representing 75% of the intended MN height.

### 3.4. Penetration Depth of the Staggered MN Arrays

**Figure 7:**
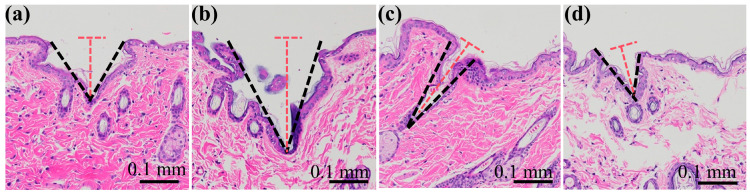
H&E staining of the rat skin penetrated by MNs: (**a**) #1 MN arrays; (**b**) #2 MN arrays; (**c**) #3 MN arrays; and (**d**) #4 MN arrays. Graphical representation: The black dotted line indicates the puncture outline, and the red dotted line indicates the puncture depth.

Skin tissue is a viscoelastic material; it deforms when punctured by MN arrays. However, the MN may not fully penetrate the skin, potentially affecting their functionality. The penetration depth of the MN into the skin depends on factors such as the MN structure and array arrangement. Figure 7 presents stained images of rat skin tissue sections that have been punctured with a MN, clearly displaying the puncture marks. These results indicate that the MNs were successfully inserted into the skin.

The staggered design of the array incorporated 12 multiplexed MNs of varying heights, leading to different penetration depths. We compared the penetration depths of the four MN arrays, using the depths of the lowest MNs as a reference point. The staggered design influenced the penetration depth, with #2 and #3 displaying significantly greater penetration compared to #1 and #4, which presented shallower penetration depths.

### 3.5. Puncture Processes of Staggered MN Arrays

**Figure 8:**
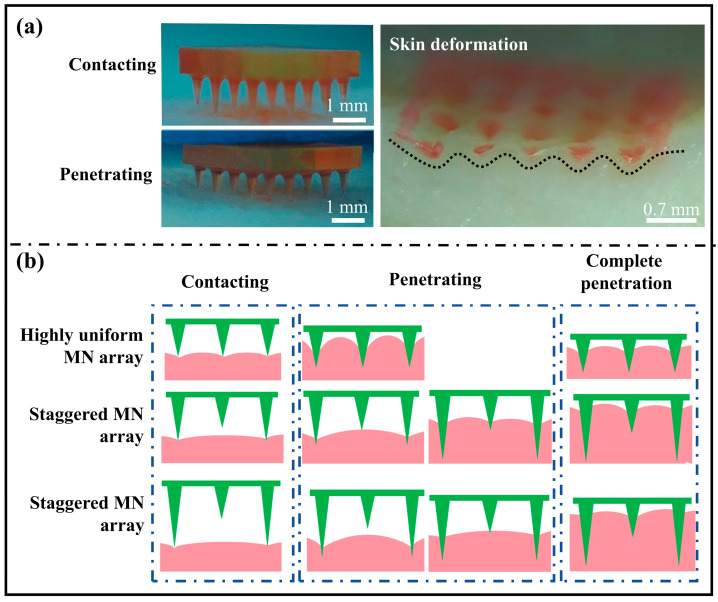
Puncture processes of staggered MN arrays: (**a**) physical pictures and (**b**) schematic diagrams.

To facilitate clear observation of the MN puncture process, paint was applied to the surface of the MNs. The painted MN arrays were subsequently pressed onto the porcine skin, as illustrated in Figure 8a. The height difference in the staggered structured MN array results in only the taller MNs contacting the skin, whereas the shorter MNs remain elevated. As the puncture process progresses, the MNs at both heights come into contact with the skin, and slight indentations appear on the surface of the skin. After the removal of the MNs after the puncture is completed, significant bumps and depressions are observed on the porcine skin.

As discussed in Section 3.3 and Section 3.4, the critical factor in designing staggered MN arrays is the height variation between these layers. Notably, the penetration capability of staggered MN arrays does not diminish indefinitely because of this height difference; instead, it remains within a defined range. By integrating the results from Figure 8a with the skin deformation data obtained from finite element analysis in previous studies [20,21], we investigated the penetration mechanism of the staggered MN arrays. Figure 8b shows a schematic diagram of conventional, highly uniform MNs and staggered MN arrays puncturing the skin. During the initial contact between the MNs and skin, the MNs cause elastic deformation of the skin, which results in a depression at the tip site and the formation of a bulge between the MNs. In a conventional, highly uniform MN array, all MNs are assumed to function identically. In a staggered MN array, the high MNs contact the skin first, and because the staggered structure is designed to increase the MNs tip spacing, the height of the bulge is determined by the spacing of the high MNs in the array, given that the depth of the indentation formed on the skin is uniform. Upon entering the puncture phase, all the MNs within the uniform MN arrays demonstrated consistent puncture characteristics. The skin bulge between the two MNs becomes more pronounced. In staggered MNs, the skin around the MNs was depressed when the higher MNs were punctured, and the skin produced a slight stretching effect. Skin tension facilitates MNs puncture, so when the height of the low MNs is right, the low MNs will take advantage of the puncture. Since the skin deformation caused by high acupuncture will recover after the skin is broken by high acupuncture, the internal stress of the skin will be released, and the height of the skin eminence between the MNs will decrease. Therefore, if the height of the low MNs is too short, these MNs will need to self-puncture without the help of the tension generated by the higher MNs.

### 3.6. Hydrophilicity of MN Arrays

**Figure 9:**
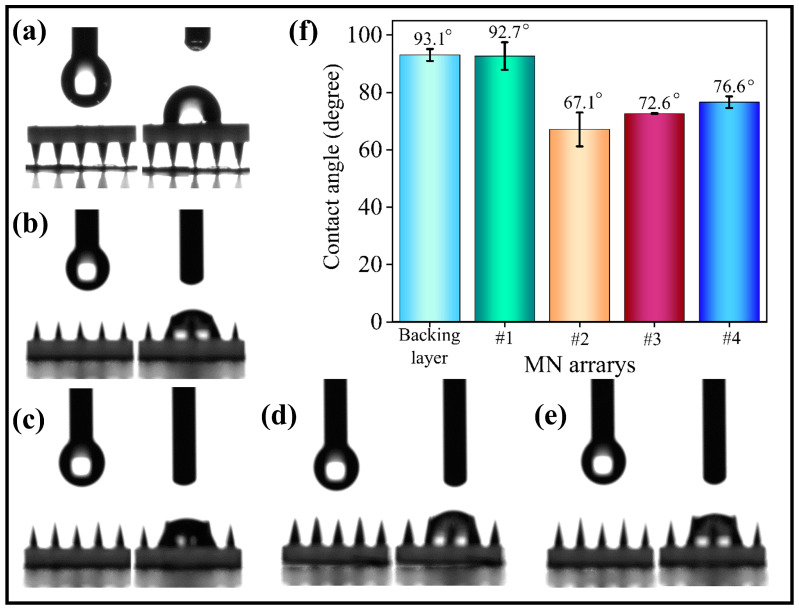
The hydrophilicity for MN arrays: (**a**) backing layer; (**b**) #1 MN arrays; (**c**) #2 MN arrays; (**d**) #3 MN arrays; (**e**) #4 MN arrays; and (**f**) contact angle of MN arrays.

MNs are classified into solid MNs, coated MNs, soluble MNs, dissolvable MNs, hollow MNs, and porous MNs, and the principles of drug delivery and monitoring associated with these MN types are well documented in the literature. Liquid–state mRNA vaccines have garnered considerable attention in the field of vaccine delivery, especially since the introduction of COVID-19 mRNA vaccines [22]. The hydrophilicity of the drug delivery MN arrays enhances the binding affinity of the liquid drug to the MN matrix. This property reduces drug loss during insertion and facilitates the absorption of subcutaneous interstitial fluid, thereby increasing drug solubilization and release [23,24]. Monitoring MNs requires more complex surface functionalization methods, including metallization, various enzyme membrane coatings, and protective coatings. Increased hydrophilicity improves the stability of these coatings and biomarker extraction [25,26]. Therefore, a preliminary evaluation of the hydrophilicity of these structures was performed. Figure 9 shows the hydrophilicity results of the four MN arrays and their backing layers (i.e., non-MN structure planes). The results indicate that the water contact angle of the MN backing layer, as well as that of the traditional highly uniform MN array, is approximately 93° ± 4°. This value is positioned between hydrophilic and hydrophobic classifications. The staggered design decreases the water contact angle to 67.1° ± 5°, 72.6 ± 2°, and 76.6 ± 2°, and most decreased amplitude to approximately 26°.

The hydrophilicity of MNs is influenced by their preparation materials and surface energy. Additionally, the hydrophilicity increases as the distribution density of the MNs decreases. The staggered structural design reduces the three-dimensional distribution density of the MNs, which in turn affects its surface energy. Consequently, this reduction in surface energy leads to a decrease in the droplet contact angle, thereby enhancing the hydrophilicity of the MN arrays.

### 3.7. Potential Application Research–Drug Release to Various Skin Layers

In this section, the potential applications of staggered-design MNs are studied, and the coated staggered MN array and hollow staggered MN array were designed. Section 3.6 shows that the 3D-printed MN arrays exhibit low hydrophilicity, which is not conducive to drug delivery. To address this issue, we performed surface treatment on the MN arrays via a plasma processor.

**Figure 10:**
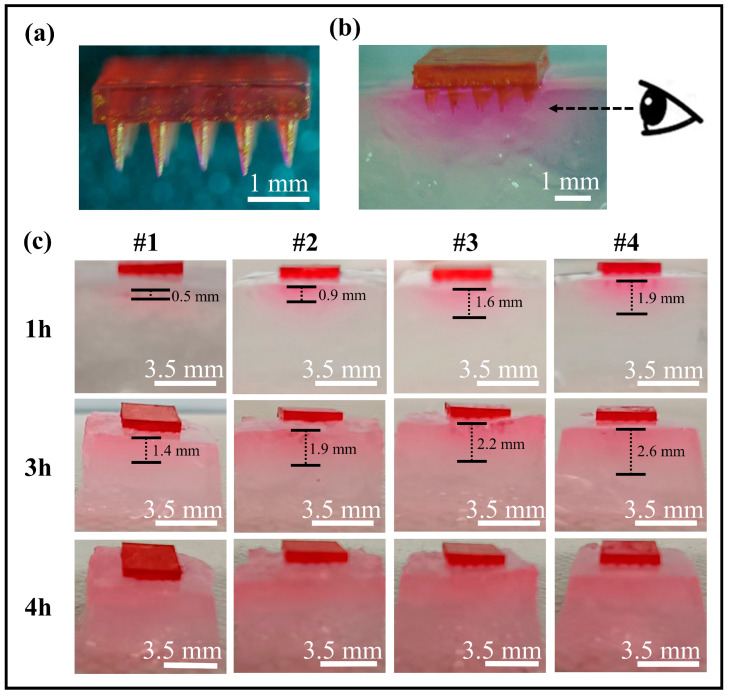
Drug release behavior of the coated MN arrays: (**a**) photographs of the rhodamine-B-coated MNs; (**b**) photographs of the released rhodamine B from the MN patches into the agarose gel; and (**c**) the color of the agarose gel containing released rhodamine B over time.

In this study, we evaluated the coated MN loading and release of hydrophilic small molecule drugs using Rhodamine B as a model compound. As illustrated in Figure 10a, the fluorescent dye Rhodamine B was dispersed in a Nafion solution and uniformly coated onto the surface of the MN arrays. The coated MNs were inserted into agarose gels, and the color changes due to drug release were monitored at various time intervals (Figure 10b).

The diffusion results are illustrated in Figure 10c. After one hour of application, the dye diffusion depth in the agarose gel measures approximately 0.5 mm, while the diffusion depths for the staggered MNs are 0.9 mm, 1.6 mm, and 1.9 mm. With the extension of the application time, the difference in the diffusion depth decreases, and the diffusion depths are 1.4 mm, 1.9 mm, 2.2 mm, and 2.6 mm when it reaches three hours. At four hours, the dye permeated the entire agarose gel. In the initial release phase, there is a positive correlation between the diffusion velocity and the length of the taller MNs in the array, wherein longer MNs spatially increase the drug release range.

**Figure 11:**
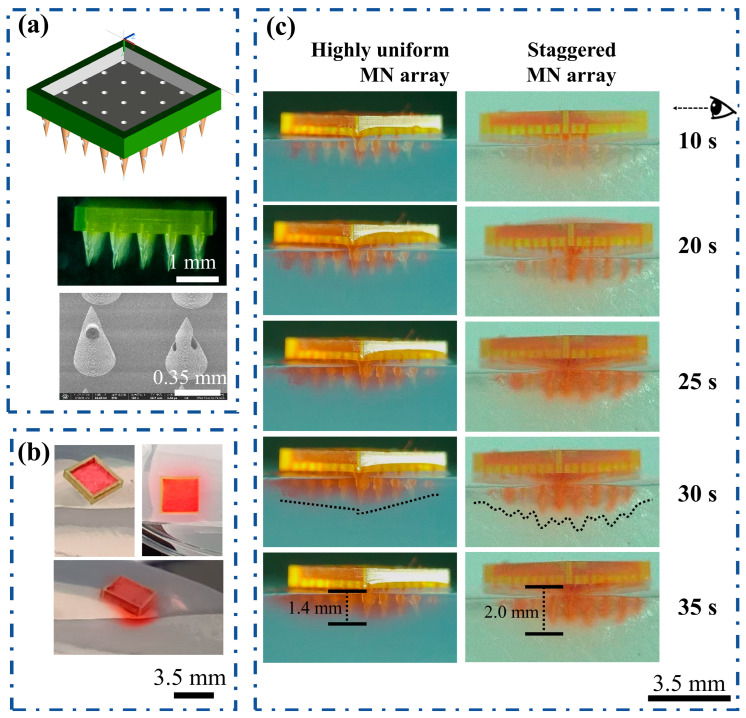
Drug release behavior of the hollow MN arrays: (**a**) morphology of the hollow MN arrays; (**b**) physical picture of the hollow MN puncture into the agarose gel; and (**c**) the color of the agarose gel over time.

In the treatment of wounds, scar tissue, burned skin, skin infections in diabetic patients, and other conditions, the properties of each skin layer vary. Layered drug administration significantly enhances skin recovery. On the basis of the results of the previous study, we continued to prepare staggered hollow MN arrays with heights of 0.8 mm and 1.0 mm. Figure 11a shows the shape of the staggered hollow MN arrays, which can be easily fabricated via a 3D printer. To clearly demonstrate the effects of layered drug delivery, we used pigments to simulate drugs. These pigments were fixed onto absorbent paper and embedded in the grooves of the MN array’s backing layer, as shown in Figure 11b.

Hollow MN arrays were inserted into agar gel to observe drug diffusion over various time periods. Figure 11c shows that the diffusion pattern of the pigment surrounding each MN in arrays of equal height was consistent, with no significant differences observed. In the hollow MN arrays featuring a staggered structural design, distinct diffusion patterns of pigments were observed around MNs of varying heights. For a diffusion time of 35 s, the diffusion depths were 1.4 mm and 2.0 mm, respectively. The hollow structure design enabled us to clearly observe the effects of the staggered structure on drug release at various positions within the agarose gel, thereby establishing a foundational design for future staggered structures.

## 4. Conclusions

This work investigated the feasibility of using 3D printing to fabricate UV-curable polymer-staggered MN arrays for transdermal layered drug delivery. The conclusions are as follows:

The fabricated staggered MN arrays exhibited morphological stability, consistent layer thicknesses, and tips measuring approximately 0.01 mm with high precision.

The fracture forces of the staggered MN arrays were 23.85 N, 6.05 N, 4.97 N, and 3.93 N, respectively. The fracture forces of the individual MNs were approximately 0.93 N, 0.53 N, 0.38 N, and 0.32 N, indicating their ability to puncture the skin.

The staggered MN arrays significantly decreased the total puncture force of the MN array, with the maximum puncture force potentially reduced by approximately 80% due to variations in the staggered height.

Investigations into the puncture depth and the puncture process revealed that the staggered MN arrays could effectively enhance the puncture depth for the lower MNs. However, this enhancement is not consistently maintained because of changes in the staggered height difference; thus, the staggered height difference should remain within a reasonable range, with an optimal height difference between 0.16 mm and 0.32 mm.

Compared with 3D-printed planar structures and highly uniform MN arrays, the staggered design altered the hydrophilicity of the MN arrays, reducing their water contact angle from 93° to approximately 70°.

3D printing technology provides a rapid preparation method for preparing coated and hollow staggered MN arrays. Drug release studies of these two structures in simulated skin have shown that the staggered design can effectively achieve transdermal layered drug delivery and improve the efficacy of MNs in the three-dimensional space of the skin.

In conclusion, compared with conventional, highly uniform MN arrays, staggered MN arrays reduce the skin penetration force, increase the penetration depth, improve the hydrophilicity of the arrays, and achieve layered drug delivery within the skin, which is expected to contribute to advancement in MN drug delivery technology. This preliminary study provides a case study for expanding the three-dimensional spatial application of MNs within the skin. In this work, only conical MNs with a height of 0.8 mm were initially investigated. Further studies need to explore the staggered design of MN arrays of different geometries and sizes, with the ultimate goal of increasing the MN density to reduce the total patch size and to improve the drug delivery efficiency.

## Data Availability

The original contributions presented in the study are included in the article, and further inquiries can be directed to the corresponding authors.
